# Relational sonic pedagogies for ocean engagement: soundwalking, soundsitting, and soundweaving

**DOI:** 10.3389/fpsyg.2026.1780797

**Published:** 2026-04-14

**Authors:** Kimberly M. Post

**Affiliations:** Department of Psychology, Northeastern University, Boston, MA, United States

**Keywords:** deep listening, geocentric ethics, nature connection, ocean engagement, ocean stewardship, sonic pedagogy, sonic relationality, soundweaving

## Abstract

This conceptual analysis proposes a triadic sonic pedagogy for relational ocean engagement, developed for educational and community-based ocean literacy and stewardship initiatives. Grounded in Pauline Oliveros's Deep Listening and Steven Feld's Sonic Relationality, it challenges extractivist imaginaries framing the ocean as passive, and builds on humanities scholarship recognizing oceanic dynamism, by emphasizing the ocean's acoustic agency in coastal and nearshore sonic practice. It develops three interconnected practices —the established practice of soundwalking, the emerging practice of soundsitting, and the novel pedagogical practice of soundweaving—that transform abstract marine concepts into lived multisensory encounters, strengthening empathy, communal bonds, and ethical stewardship. These scalable practices can be used sequentially within a single session or adapted flexibly to different contexts, drawing on multiple cultures and traditions to support careful, attuned engagement with ocean soundscapes. By centering the ocean's acoustic agency, this model challenges anthropocentric paradigms, positions sound as a portal for collective care, and contributes to ocean literacy, geocentric ethical awareness, and relational forms of ocean citizenship in a climate-challenged world.

## 1 Introduction

The ocean, as both a physical and cultural presence, invites unique possibilities for engagement through sound as an emergent vector for connection, meaning, and care (Abels and Eisenlohr, 2025; Fisher, 2024; Giomi, 2019; Helmreich, 2015; Steinberg and Peters, 2015; Whittaker, 2024, 2026; Whittaker et al., 2024, 2025). This conceptual analysis proposes a triadic sonic pedagogy—soundwalking, soundsitting, and soundweaving—grounded in Deep Listening (Oliveros, 2005) and Sonic Relationality (Feld and Boudreault-Fournier, 2019) to reimagine ocean engagement as a geocentric relational practice. While building on ocean sound work that already treats the sea as dynamic and relational through recordings and mediated listening (Helmreich, 2015; Whittaker, 2024, 2026; Whittaker et al., 2024, 2025; Steinberg and Peters, 2015), the triad's distinctiveness lies in its emphasis on real-time, *in-situ* listening and carefully framed co-creation with live coastal and nearshore ocean soundscapes, including hydrophone streams. In this sense, the model does not claim exclusivity in challenging extractivist framings, but offers a specific pedagogical pathway that centers embodied, place-based sonic encounter.

Listening with and through the ocean brings attention to the complex web of relationships that flows across human and more–than–human worlds, centering it not as a passive backdrop, but as a dynamic place of encounter, relationality, and transformation. This approach resonates with work in ocean studies and human geography that reconceptualizes the sea as a dynamic, volumetric, more–than–wet space shaped by entangled human-ocean relations rather than as an empty surface or passive resource (Anderson and Peters, 2014; Steinberg and Peters, 2015; Peters and Steinberg, 2019). Approaching the ocean in this way enacts a geocentric ethics, in which responsibility and attentiveness extend beyond human interests to include the more–than–human relations that constitute marine worlds. Recent scholarship frames ocean literacy as a multidimensional construct—not merely conceptual knowledge, but encompassing awareness, behavior, emotional connection, access, experience, and relational dimensions across contexts (McKinley et al., 2023; Bastos et al., 2025; Strand et al., 2022). These frameworks provide generative guidance for inclusive, multisensory pedagogies that invite understanding of the ocean not merely as a resource but as a sonic participant in place–based learning and collective practices of care within specific educational and community contexts (Abels and Eisenlohr, 2025; Fisher, 2024; Giomi, 2019).

This article is written primarily for educators, facilitators, and practitioner–scholars working in education, community programs, and informal ocean engagement settings who seek relational, multisensory approaches to ocean literacy and stewardship, while remaining accessible to practitioners interested in applying sonic pedagogies in their own contexts. It advances a conceptual pedagogical framework for relational ocean engagement rather than reporting empirical findings, and it treats soundwalking, soundsitting, and soundweaving as adaptable practices that can be incorporated into courses, workshops, and public engagement initiatives, and optionally used as methods within research and community projects. Across these contexts, the triadic model is offered as a way to design learning experiences that center relational listening, multispecies attunement, and collective responsibility within ocean spaces.

In this framework, coasts are approached as liminal oceanic zones where marine, terrestrial, and atmospheric processes audibly converge, making ocean soundscapes experientially accessible as “more-than-wet” interfaces rather than as a sharp boundary between land and sea. The triad therefore focuses on coastal and nearshore ocean spaces, including hydrophone-mediated subsurface sound, as primary sites for practice, while remaining in dialogue with offshore and deep-water acoustic research. This situated emphasis acknowledges both the practical constraints of in-person sonic pedagogy and the conceptual importance of coasts as zones where human-ocean relations are lived, negotiated, and heard.

In what follows, ocean literacy is understood not only as familiarity with marine systems and processes, but as a situated capacity to listen to and interpret ocean soundscapes as part of socio-ecological life (McKinley et al., 2023; Bastos et al., 2025). Geocentric ethics draws from ecological philosophy and more-than-human ethical traditions that decenter the human and emphasize Earth-centered responsibility (Abram, 1996, 2010; Bateson, 1979; Kimmerer, 2013; Todd, 2016), and here it is approached through practices that emphasize the ocean's acoustic agency and more-than-human relations. Relational citizenship signals forms of ocean engagement grounded in mutual responsiveness, shared responsibility, and community practices of care rather than solely individual knowledge acquisition or behavior change (Gould et al., 2021; Kimmerer, 2013).

The triadic model brings together soundwalking, soundsitting, and soundweaving because, in combination, they trace a deliberate pedagogical progression from initial sensory attunement to co–creative relational practice. Soundwalking introduces participants to coastal and marine soundscapes through mobile, observational listening, building foundational awareness and curiosity. Soundsitting extends this awareness temporally and relationally by inviting longer–term, situated listening and shared reflection in specific ocean places. Soundweaving then builds on these capacities by asking participants to respond sonically with humility and care, co–creating with ocean soundscapes and one another. Taken together, the triad offers more than the sum of its parts: it provides a scaffolded pathway from noticing and naming ocean sounds, to dwelling with their temporal and affective dimensions, to enacting relational forms of ocean citizenship through co–creative sonic practice.

Pedagogically, the triad is designed to support a sequence of learning and transformation. Soundwalking cultivates perceptual acuity, place–based awareness, and initial curiosity about coastal and marine soundscapes. Soundsitting then deepens this awareness into sustained, reflective engagement, highlighting temporal change, affect, and shared interpretation in specific ocean places. Soundweaving invites participants to translate these listening capacities into co–creative practice, experimenting with how they might respond to and collaborate with ocean soundscapes and one another. Across the triad, the emphasis is on shifting from detached observation toward relational ways of knowing and acting, in which ocean literacy, geocentric ethical awareness, and relational ocean citizenship are experienced and rehearsed through embodied listening practices.

## 2 Deep listening

The term *deep listening* was first articulated by composer and sound pioneer Pauline Oliveros in connection with her 1988 *Deep Listening* recording, and subsequently across pedagogical, philosophical, and activist contexts, to highlight the distinction between the automatic process of hearing and the intentional practice of listening (Williger, 2020). Oliveros taught that, while hearing is a passive, involuntary, and continuously occurring act, listening demands a conscious, deliberate attention that cultivates heightened awareness of both external environments and internal states (Daughtry, 2023; Oliveros, 2010; Ristić, 2021). Oliveros later described deep listening as a form of “radical attentiveness”—a dyadic, embodied openness to the present moment not limited to the ears, but engaging the whole body, memory, imagination, and empathy (Oliveros, 2005, 2010).

The roots of deep listening can be found in Oliveros (1974) work *Sonic Meditations*, where she created participatory exercises designed for communal engagement and the dismantling of hierarchical structures within performance. Sound was reimagined not as a one-directional transmission from performer to audience, but as an expanding project of collective awareness and presence. This foundation led Oliveros to further articulate deep listening as an embodied approach that invites participants into the fullness of sonic environments, embracing every sound, silence, and resonance to cultivate expanded consciousness and a sense of ecological responsibility (Aftandilian, 2022; Oliveros, 2005).

Over time, deep listening has become an ethos, “an awareness of and attention to the harmonic interconnectivity of all beings and objects” (Lipari, 2014, p. 2–3). As a form of attunement beyond heightened perceptual acuity, the practice transcends the technical or aesthetic, cultivating ethical relationality and centering reciprocal care, attentiveness, and openness across the boundaries of the human and more-than-human world (Abram, 1996, 2010; Bjelica, 2022; Lipari, 2014).

In ocean contexts, deep listening becomes a way of entering into relation with marine soundscapes as living, more–than–human environments, emphasizing the ocean's acoustic agency and inviting listeners to experience responsibility and care through continuous, embodied attention to sea–borne sound. Here, the ocean's “acoustic agency” does not imply human-like intentionality, but rather enacts how oceanic processes and more-than-human beings actively constitute sonic environments that call forth, shape, and invite human listeners into relational co-presence and response through embodied attention to marine acoustic environments.

## 3 Sonic relationality

Anthropologist Steven Feld articulated the concept of *sonic relationality* through his pioneering work on acoustemology, a term he uses to describe a way of knowing and being in the world that emphasizes relationality through sound (Feld, 1996). In asking what might be understood by attending to the “sonic relationality of human voices to the sounding otherness of presences and subjectivities like water, birds, and insects” (Feld, 2017, p. 93), he challenges traditional subject-object paradigms by emphasizing subject-subject relationships enacted in and through sonic engagement. In this way, sonic relationality becomes a practice of reciprocal connection rather than detached observation.

According to Feld, sonic relationality demonstrates that knowing the world through sound is inseparable from inhabiting it sonically; listening becomes an embodied, participatory, and situated practice (Feld, 2021). Sound thus functions not simply as an object of analysis but as a dynamic, emergent force that creates and reflects social and ecological relationships (Chung, 2021; Feld, 2021). In Feld's acoustemological framework, sonic relationality encourages modes of listening attuned to layered meanings and interactions, recognizing the agency of more-than-human actors and—with Batesonian focus on the intertwined sociality of nature—the sensory, cultural, and ecological contexts that shape sonic experience (Bateson, 1979; Feld, 2012).

In ocean contexts, this attention to sonic relationality converges with work on marine soundscapes and ocean acoustics, which use hydrophone recordings and soundscape analysis to understand how ocean spaces emerge through interacting biophony, geophony, and anthropophony—and how listening can inform both science and stewardship (Duarte et al., 2021; Miksis-Olds et al., 2018). This work is primarily oriented around natural science questions and the production and analysis of acoustic data about marine ecosystems. By contrast, the triadic model developed here approaches ocean soundscapes as sites of human-ocean relationality, drawing on sound studies and the environmental humanities to complement these scientific approaches with pedagogical practices of listening, co-presence, and care. Anthropological research on ocean sound has likewise explored how listening to underwater recordings reshapes understandings of life, environment, and the limits of the human (Helmreich, 2015). Thinking about sonic relationality in these ocean contexts complements ocean–space perspectives by suggesting that listening is not only a way of knowing marine environments, but a way of inhabiting ocean space as fluid, voluminous, and relational, in line with calls to understand oceans as more–than–wet, lived spaces rather than distant backdrops (Steinberg and Peters, 2015; Peters and Steinberg, 2019).

When grounded in immersive sound-based pedagogy, deep listening and sonic relationality each provide robust frameworks for ocean engagement that encourage a reimagining of the ocean as an active collaborator. Through these frameworks, three distinct practices—the established soundwalking (Schafer, 1977; Westerkamp, 1974), emerging soundsitting (Bruce, 2023), and soundweaving (introduced here as a novel pedagogical method)—function as immersive, community-based pedagogies facilitating dialogic encounters between participants and ocean environments (Strand et al., 2022). These nurture shared responsibility and collective care within specific educational and community contexts.

## 4 Soundwalking

In the triadic framework, *soundwalking* functions as an introductory, mobile practice that cultivates observational listening and foundational sensory knowledge of coastal and marine soundscapes. The term was first widely popularized by composer and acoustic ecologist R. Murray Schafer in the late 1960s as part of the World Soundscape Project at Simon Fraser University (Schafer, 1977). Schafer introduced soundwalking as an empirical method to identify and analyze soundscapes and their components in various environments (Adams et al., 2008). However, the practice itself is primarily attributed to acoustic ecologist Hildegard Westerkamp, a key member of the World Soundscape Project, who defined soundwalking as “any excursion whose main purpose is listening to the environment” (Westerkamp, 1974). Since its inception, soundwalking has developed into a well-established practice grounded in scholarly literature, especially within sound studies and acoustic ecology, where it has been employed as an educational tool to enhance sonic awareness and listening skills (Behrendt, 2018).

Evolving from Schafer's methodological foundations, soundwalking is characterized by mindful immersion and focused listening to cultivate acute awareness of the sonic environment and its constituent sounds (Bitter, 2022). This embodied and intentional engagement with soundscapes deepens the listener's sonic connection to place, contributing to sensory ethnographies and environmental understanding (Davies et al., 2009; Stevenson and Holloway, 2017). Soundwalking operates both as an artistic practice and a research methodology, encompassing diverse modes of multisensory listening and attuning individuals to their acoustic surroundings. In sustainability and conservation contexts, soundwalking has been employed to document threatened soundscapes through field recordings of endangered habitats and to raise awareness of environmental issues such as noise pollution and biodiversity loss (Challe, 2015; Gale et al., 2025).

Soundwalking within the context of ocean engagement is a participatory and communal practice that promotes attentiveness to coastal and marine soundscapes while nurturing collective care for the ocean environment. By mindfully walking along shorelines, estuaries, or tidal zones, participants listen deeply to the intricate acoustic ecology—the interplay of waves, seabirds, wind, and human activity—experiencing multisensory attunement to place (Music for the Sea, 2024; Ocean world of Sound, 2023). This embodied practice not only enhances awareness of diverse sound sources but also supports relational understanding through shared experience and dialogic exchange among participants. As an approach to ocean pedagogy, soundwalking facilitates the co-creation of knowledge and community stewardship rooted in reciprocal listening and ecological sensitivity. In this way, soundwalking supports ocean literacy by transforming abstract knowledge about marine environments into situated, sensory understanding of specific coastal and marine soundscapes, aligning experiential listening with broader calls to approach ocean literacy as a multidimensional construct rather than solely a body of facts (McKinley et al., 2023). For many participants, this shared listening can evoke emotions such as awe, calm, grief, or unease as they encounter both the beauty and disruption in marine soundscapes, and these feelings often become a basis for renewed relational commitment to particular ocean places and communities (Severin et al., 2022).

Initiatives such as *Posidonia Soundscapes* in the Mediterranean (Music for the Sea, 2024) utilize guided soundwalks and immersive listening journeys as innovative methods to bridge scientific knowledge and community engagement. These soundwalks invite participants to move mindfully through marine environments with heightened sensory awareness, and encourage co-creation of meaning through storytelling, reflection, and creative response (Barclay, 2017; Stevenson and Holloway, 2017). Central to this practice is the concept of ethical listening as an act of relationship-building—listening not merely for information but as an embodied, reciprocal dialogue with marine ecosystems (Barron and Losleben, 2025; Music for the Sea, 2024).

Soundwalking cultivates community by making audible the often-invisible sonic dimensions of the ocean, connecting diverse stakeholders—including scientists, residents, fishers, artists, and policymakers—through shared listening experiences (Music for the Sea, 2024; Williams, 2017). This multisensory engagement provides direct experiential access to marine soundscapes encompassing biotic sounds, abiotic processes, and anthropogenic influences that tend to be overlooked in mainstream environmental discourse (Miksis-Olds et al., 2018). By centering collective attention on these acoustic ecologies, soundwalking transcends disciplinary and cultural boundaries and promotes dialogic exchange, interdisciplinary knowledge co-creation, and collaborative stewardship. This embodied attentiveness anchors abstract environmental phenomena—such as biodiversity changes, noise pollution, and ecosystem health—in lived, relational experiences, inviting collaborative dialogue informed by sensory engagement rather than detached observation (Cooke and West, 2016; Flood et al., 2021).

Through engagement and reflection, soundwalks transform abstract environmental phenomena into lived realities, anchoring ocean care in relational and experiential contexts (Barclay, 2017; Stevenson and Holloway, 2017). This grounding cultivates empathy and deepens social cohesion oriented toward ecological responsibility, reinforcing the tenets of deep listening and sonic relationality frameworks that emphasize ongoing relational practices embedded in place and community (Feld, 2017; Oliveros, 2010; Music for the Sea, 2024; Westerkamp, 2021). Soundwalking as a learning practice encourages sensory acuity, place–based awareness, and initial curiosity about coastal and marine soundscapes, laying a foundation for more sustained and co–creative forms of ocean engagement (Galloway, 2015).

While soundwalks have significantly advanced soundscape research through direct, embodied encounter with environmental acoustics, they also present limitations. Participants typically listen silently during walks and reflect afterward, which can hinder real-time dialogue and shared interpretation (Bruce, 2025). They are also commonly single, short-term events lasting 30 to 60 min, that capture only transient snapshots of sonic environments and are limiting to sustained engagement with dynamic soundscapes (Stevenson and Holloway, 2017). In ocean contexts, soundwalks are further constrained to what can be heard from walkable coastal areas, excluding many sub-surface and distant marine sounds that become accessible only through hydrophones and recorded ocean audio. Ocean soundscape research has shown how such recordings can reveal the complex mix of biotic, abiotic, and anthropogenic sounds that shape marine environments, and how listening to these soundscapes can inform understanding of biodiversity, ecosystem health, and human impacts (Miksis-Olds et al., 2018; Duarte et al., 2021). Soundwalking can also be restrictive for those who are unable to walk, require modified access for walking, or use mobility aids, underscoring the importance of adapting soundwalking methods for diverse mobilities (Bruce, 2023; Mediastika et al., 2022; Paquette and McCartney, 2012).

These limitations do not diminish soundwalking's foundational role within the triad; rather, they clarify its specific pedagogical contribution as an opening practice that cultivates sensory acuity, place-based awareness, and initial curiosity about coastal and marine soundscapes. Soundwalking's mobile, observational listening provides the perceptual foundations essential for soundsitting's sustained temporal dwelling and soundweaving's co-creative engagement, so that together these practices form mutually reinforcing pathways from sensory awakening to relational ocean citizenship rather than sequential replacements.

## 5 Soundsitting

*Soundsitting*, a term first used by researcher Bruce (2023), extends soundwalking in the triadic framework by deepening temporal and situated receptivity, enabling participants to dwell with ocean soundscapes over time and to reflect together on their emerging perceptions and emotions. It offers a nascent, complementary practice to soundwalking that addresses the accessibility concerns of the latter through stationary, contemplative listening over extended periods. Because it is stationary, soundsitting can be practiced on shorelines, docks, vessels, or even in inland spaces via hydrophone streams and marine recordings, bringing participants together “within both a space and a process that allows [them] the time to consider, reflect, and discuss their listening experiences as they unfold, and to do so recurrently over an extended period” (Voegelin et al., 2022; Bruce, 2023, p. 6). Both soundwalking and soundsitting seek to deepen awareness of place by revealing the subtle and often overlooked qualities of sonic environments; through attentive listening, these methods enable participants to develop a richer understanding of their surroundings and the communities embedded within them (Barclay, 2017; Bruce, 2023; Stevenson and Holloway, 2017).

The *Biosphere Soundscapes* project provides a large-scale example of soundsitting-inspired practice. Taking place in several globally significant biosphere reserves including the Noosa Biosphere Reserve in Australia and the Tonle Sap Biosphere Reserve in Cambodia, it is an interdisciplinary project that explores the value of acoustic ecology in understanding rapidly changing environments. The project works with artists, scientists, research laboratories, and local communities, employing long-term, stationary, immersive listening sessions *in situ* with extended temporal engagement through repeated listening sessions and community-based sound workshops (Barclay, 2017). This approach produces a rich, dialogic relationship with changing ecosystems, allowing participants to perceive ecological rhythms that are overlooked by episodic or mobile listening methods. Listening sessions and community workshops are combined to generate sensory, cultural, and ecological knowledge that promotes conservation and social justice. Extended longitudinal engagement helps reveal subtle acoustic shifts and facilitates collective environmental care practices rooted in cultural meaning and place-based knowledge (Barclay, 2017).

By cultivating prolonged, recurrent listening in place and inviting collective reflection, soundsitting deepens ocean literacy as a temporal and relational capacity, attuning participants to ecological rhythms, change, and vulnerability in particular marine environments. This emphasis on temporal immersion, shared reflection, and place-based listening resonates with emerging framings of ocean literacy that highlight relational experience, affect, and ongoing engagement rather than one–off informational encounters (Bastos et al., 2025; McKinley et al., 2023; McRuer et al., 2025).

Rousell and Peñaloza-Caicedo (2022) present an innovative model of immersive, speculative listening practiced along Birrarung Marr, a culturally and ecologically significant river park in Melbourne, Australia. This project moves beyond conventional soundwalking by centering prolonged, seated listening sessions where participants experience sustained sensory integration with the evolving acoustic ecology of the river environment. Participants engage in slow, focused listening to live river sounds—including water flow, wildlife calls, human activity, and the seasonal shifts—within a framework that integrates Indigenous cosmologies, reflection, and critical awareness of urban environmental dynamics. The stationary format of soundsitting allows experiences of layered temporalities—tidal cycles, diurnal shifts, seasonal rhythms, and subtle ecological changes—within river and ocean soundscapes that would be difficult to capture during brief mobile listening events (Rousell and Peñaloza-Caicedo, 2022). These dimensions invite participants to dwell with environmental changes, uncertainty, and vulnerability rather than treating soundscapes as static snapshots.

The project also incorporates dialogic knowledge production through facilitated sharing circles where participants collectively reflect on their embodied listening experiences. In this specific context, iterative community engagement anchors relational environmental care in Indigenous place-based knowledge (Rousell and Peñaloza-Caicedo, 2022). Following Haraway (1988) notion of situated knowledges, such practices must always emerge from local histories, communities, and more-than-human relations rather than importing epistemologies from other contexts—attending carefully to whose knowledges are accountable to place.

Immersive soundsitting supports ongoing engagement with environmental change and uncertainty, encouraging participants to “listen for futures” attentive to loss, resilience, and collective responsibility (Rousell and Peñaloza-Caicedo, 2022). This intensifies emotional encounters with slower temporalities that surface sorrow, anxiety, or hope, while collective formats hold these affects relationally (Carasso Romano et al., 2025; Pihkala, 2025; Severin et al., 2022). Pedagogically, soundsitting cultivates patience and emotional attunement, supporting learners in dwelling with oceanic change rather than seeking quick resolution (Bruce, 2023).

## 6 Soundweaving

Within the triadic model, *soundweaving* culminates as a co-creative practice where participants braid their own sounds into marine and coastal soundscapes, entering felt, situated relationships with ocean environments and one another. As framed here, soundweaving extends the relational immersion of soundwalking and soundsitting by inviting real-time sonic co-composition with the more-than-human world. Though not yet implemented in formal ocean engagement programs, it proposes a pedagogical model guided by explicit ethical constraints—responsiveness, spaciousness, and non-interference—to cultivate relational ocean literacy rather than artistic display or scientific communication.

Soundweaving shares affinities with ecoimprovisation and site-responsive sound art (Hayes, 2017; Aliel et al., 2024; Whittaker, 2024), yet remains distinct through deep listening principles (Oliveros, 2005) and non-hierarchical dynamics. In ocean contexts, such co-creation generates “submersive affective atmospheres” (Whittaker et al., 2025), drawing participants into volumetric more-than-wet worlds. Responding to sonic textures like wind, waves, or wildlife, participants experience humans as co-creators within a larger sonic tapestry—encouraging reciprocity, humility, and place-attunement in alignment with Oliveros' sonic embodiment (2005) and Feld's (2012) participatory sound-making.

Historical and contemporary examples of humans co-creating with animals and environments illustrate soundweaving in practice. Cellist Beatrice Harrison's early 20th-century “duets” with nightingales, broadcast by the BBC from her garden, framed the bird as an improvising partner whose song shaped and was shaped by the cello's response, drawing widespread public attention to the vitality of more-than-human musical agency (Harrison, 1927; Kennedy, 2023). Folk singer Lee's (2021a,b) *Singing with Nightingales* gatherings extend this relational ethos into participatory night walks where small groups listen in silence before carefully joining their own voices and instruments to the birds' songs, treating the encounter as a communal ceremony of listening and response (Vaughan-Lee and Lee, 2021). Similarly, soprano saxophonist Paul Winter's Earth Music project plays “ecological jazz” that blends world, classical, and jazz music with marine mammals, wolves, and birds to promote biodiversity and inspire environmental awareness (Music for the Earth, n.d.; Winter, 1990, 1993).

This co-creation of sonic environments through reciprocal attunement with more-than-human soundscapes appears across diverse traditions worldwide, reflecting longstanding relational ontologies where sound serves as a medium for communal entanglement with place, kin, and ecology. While many such practices predate colonial documentation and continue to evolve beyond Western frameworks, there are contemporary Indigenous examples that illustrate how soundweaving manifests in many contexts, offering models for ocean pedagogy rooted in collectivity and multispecies reciprocity. In the Pacific, traditional Māori practices like waiata moana (ocean songs) and haka performed during whale strandings or sea voyages entwine human voices with marine soundscapes, honoring moana (ocean) as kin through call-and-response rituals that listen to and echo whale songs and waves without technological mediation (McLean, 1996; Mika, 2021). Native Hawaiian *oli* on Hokule'a voyages blend voices with waves, winds, and pu calls, reviving *mo'olelo* and *moana* relationality as ethical ancestral practice (Diettrich, 2018; Kunisue, 2014). Diettrich (2018) documents Pacific Island musical exchanges where chants dialogically interweave with environmental cues during voyaging rituals, while Kunisue (2014) examines sonic embodiment in Polynesian wayfinding, emphasizing oli as co-creative responses to sea rhythms. These practices are grounded in Hawaiian cosmology, where human voices attune to ocean agency for kinship and care (Gould et al., 2021; Pisciotta, 2023). In each case, soundweaving emerges as a co-created sonic fabric in which human expression braids into more-than-human soundscapes.

As a pedagogical practice for ocean awareness, soundweaving—defined here as participatory co-composition with live human and more-than-human ocean soundscapes—can be introduced through sessions that invite communities to co-compose with marine and coastal acoustics. Unlike the studio-based textile-to-score mappings of the same name (Frearson, 2015; Medekšaite, 2016), this soundweaving centers real-time sonic responsiveness and non-interference within marine ecologies.

Either standalone or building on soundsitting/soundwalking, participants first attune to layered acoustic ecologies—waves, seabirds, invertebrates, boat traffic, subsurface vocalizations (Bruce, 2025)—then add simple tones, chants, body percussion, or instrumental gestures that thread: into the environment. Three ethical constraints guide this practice: responsiveness (adjusting to oceanic and human others), spaciousness (leaving room for environmental sound), and non-interference (avoiding sonic domination).

Soundweaving enacts relational co-collaboration, echoing Rights of Nature decisions like Los Cedros, Ecuador in 2021 (Eco Jurisprudence Monitor, n.d.) and Amazon region, Colombia in 2018 (Eco Jurisprudence Monitor, 2018), where nature holds legal agency. Contemporary music follows suit, crediting “NATURE” as co-composer—as in Yann Tiersen's 2025 Earth Day release “feat. NATURE” and 30+ artists adopting this precedent (Sounds Right, 2025).

These examples frame more-than-human agency as mutual entanglement, positioning the ocean as sonic co-collaborator that conditions ethical human response. Facilitators operationalize this through non-interference (never dominating marine sounds) and “consent-by-withholding”—adding human sound only when it harmonizes without overwhelming more-than-human voices.

Thus soundweaving becomes “singing with” the ocean—and each other—encouraging felt reciprocity in marine worlds, communal bonds via mutual sonic dialogue, and collective care from local advocacy to policy influence (Barclay, 2017; Rousell and Peñaloza-Caicedo, 2022). Subsequent reflection circles, journaling, or creative responses articulate shifts in understanding marine life, vulnerability, and shared responsibility. These practices invite relational ocean citizenship as an ongoing, situated participation in caring for marine places and communities (Strand et al., 2022).

This emphasis intersects growing art-science and sonic work connecting science, society, and sea through sound and improvisation (Whittaker, 2024, 2026; Whittaker et al., 2024), alongside Arctic soundscape mediation that furthers affective polar relations (Coutu et al., 2024).

Soundweaving advances relational sonic pedagogy by:

1. Framing co-creative ocean sound practices as scaffolded pedagogy rather than performance or outreach
2. Articulating progression from perceptual attunement → temporal dwelling → ethical co-creation
3. Providing concrete session structures and ethical guidelines (non-interference, consent-by-withholding) for relational ocean literacy

Participation demands heightened vulnerability—listening closely before sounding, negotiating when to contribute, stay silent, or avoid dominating the shared sonic field. Emotions like trust, humility, joy, and discomfort co-produce shared affects that deepen belonging within more-than-human ocean communities. This emotional reflexivity aligns with Levi and Peters (2024), who position affects as vital to human-ocean relations beyond technocratic governance. Soundweaving thus offers a space to rehearse relational ethics and collective responsibility together.

Soundweaving sessions unfold in four phases tailored for ocean contexts:

1. Deep attunement (10–15 min): silence or soundsitting with live coastal/hydrophone feeds, noting ocean rhythms and affects;
2. Responsive imitation (10 min): Echo wave pulses or seabird calls via voice/breath, practicing restraint;
3. Interlocking weave (15–20 min): participants layer sounds in cycles, adjusting to ocean/peers;
4. Reflective integration (10 min).

Drawing from Indonesian gamelan—traditional ensemble music of interlocking patterns and communal attunement—these phases cycle as participants grow attuned to sound, process, and each other (Lindsay, 1992; De Silva, 2025). They mirror gamelan's non-hierarchical rehearsal dynamics, cultivating communal humility across all skill levels.

Gamelan principles embody through:

- Interlocking sonic roles: individual voice/breath patterns weave into collective soundscape
- Cyclical layering: repeated ocean motifs build emergent harmony over iterations
- Dynamic attunement: participants adjust volume/timing to ocean and peers

Soundweaving is ideally preceded by soundwalking/soundsitting, which establish participants' perceptual foundations (awareness of marine soundscapes) and knowledge foundations (temporal/ecological rhythms) essential for reciprocal co-creation. While each practice can stand alone—soundwalking developing sonic awareness, soundsitting temporal sensitivity, soundweaving co-creative ethics—they lack preceding foundations when isolated (for instance, soundweaving without soundwalking misses baseline marine soundscape familiarity). This progression mirrors relational ontology: soundwalking awakens *perceptual reciprocity* with more-than-human soundscapes; soundsitting cultivates *temporal co-becoming* with ocean rhythms; soundweaving enacts *ethical co-creation* through sonic exchange. Together, they scaffold from sensory awakening to ocean citizenship.

### 6.1 A soundweaving model

Drawing from this author's experience leading gamelan-inspired deep listening workshops modeled on Oliveros (1974) *Sonic Meditations*, gamelan offers a powerful model for soundweaving's collective listening, responsiveness, and non-hierarchical collaboration. Its intricate interlocking parts demand responsive synchrony, prioritizing collective sound over individual virtuosity (Becker, 1993; Brinner, 1995; Lindsay, 1992; De Silva, 2025). This cultivates cohesion and deemphasizes ego, aligning with relational environmental practices (Woodruff, 2015). Yet experiences remain subjective—some encounter discomfort rather than cohesion, underscoring sonic pedagogies' need for diverse subjectivities.

Unlike Western jam sessions emphasizing virtuosic improvisation—like Whittaker's (2024) Ocean Science Jam for science communication—soundweaving centers non-virtuosic participation and geocentric restraint. Live marine soundscapes become co-present sonic partners, not backdrops.

Key gamelan principles translate directly:

- Aural transmission: Like Oliveros' cistern recording amplifying subtle shifts (Williger, 2020), soundweaving tunes into oceanic micro-changes (Brinner, 2010).
- Interlistening: Lipari's (2014) concept of reciprocal ensemble awareness parallels soundweaving's ocean-group attunement.
- Inclusive structure: Novices participate alongside experts, modeling multispecies mutual respect.

### 6.2 Practical applications to ocean contexts

Gamelan's interlocking patterns translate directly: participants contribute complementary tones weaving with waves, seabirds, and each other, creating emergent sonic tapestries where no voice dominates (Gold and Brinner, 2007). Call-and-response echoes marine rhythms; dynamic balance preserves spaciousness for environmental sounds (Lipari, 2014).

Sample 90-min session (coastal setting with hydrophone): participants begin seated in silence as facilitators stream live feeds of sea lion barks, wave surge, fish drumming/grunting, and perhaps the rhythmic thrum of vessel engines. After 10 min deep attunement, individuals may echo with finger percussion or hum wave undertones on breath. The group then gradually layers voice, body percussion, and simple instruments—matching tidal rhythms while responding to marine sounds and each other.

Introducing Gamelan culture, values, and methods: prior to soundweaving sessions, gamelan is contextualized through a 10–15-min interactive orientation that honors its Indonesian cultural roots and relational ethos, making it accessible to novices while aligning with deep listening principles. Facilitators begin with a brief verbal overview of gamelan's communal origins, with emphasis on its non-hierarchical structure, where interlocking parts encourage mutual attunement and humility, as in Javanese or Balinese ensembles (Lindsay, 1992; De Silva, 2025; Brinner, 2010). Participants engage hands-on with simple demonstrations, paired with a description of gamelan's role in village ceremonies, where it promotes values like communal cooperation and patient listening. This oral-aural transmission, which mirrors gamelan pedagogy itself, equips diverse groups (such as students and community members) to adapt these methods ethically to ocean contexts, threading human voices into marine soundscapes without cultural appropriation. No notation is used; instead, facilitators model restraint and responsiveness, inviting questions to co-create shared understanding.

Ethical distinction from western Jam sessions: unlike Western jam sessions—often driven by soloist virtuosity and competitive improvisation—soundweaving upholds non-hierarchical relational ethics through deliberate restraint. Participants listen extensively before contributing and prioritize cyclical attunement over individual display (Brinner, 2010; Lipari, 2014). This gamelan-inspired structure preserves co-creative humility and geocentric ethics, ensuring human voices braid supportively into marine soundscapes rather than dominating them.

Instrument selection for soundweaving: instruments or found objects of any material (recycled plastics, shells, stones, body parts, water glasses, rainsticks, frame drums, etc.) enable authentic expression for all participants while forging multisensory ocean connections. Rainsticks mimic wave hiss, drums echo tidal pulses, while a plastic bottle half-filled with pebbles emulates geophony; body percussion and voice remain primary. These choices prioritize accessible timbre over technique, welcoming novices across abilities and embodying geocentric ethics—human sounds blend humbly with marine soundscapes using whatever sustains reciprocity without domination (Oliveros, 2005).

Participant experiences typically shift from initial self-consciousness (“*My voice feels intrusive”*) to mutual entrainment (“*The ocean pulled us into its tempo”*), culminating in Oliveros-style reflections like “*I felt the sea listening back”*—echoing her radical attentiveness to sonic presence (Oliveros, 2005). Novices gain confidence through facilitators modeling restraint, entering only when ocean sounds invite space, while real-time changes like boat noise demand constant recalibration.

Participant diversity and group composition: soundweaving scales flexibly for 4–20 participants from diverse backgrounds, such as university students, community members, scientists, artists, fishers, elders (skill levels: complete novices to trained musicians). No prior experience required.

Inclusivity features:

- Mobility: stationary option complements soundwalking for accessibility.
- Age/ability: body percussion/voice for all; simple instruments optional.
- Hearing: visual cues (hand signals), tactile feedback (vibration from drums), facilitators check in visually.
- Introvert/extrovert: voluntary entry protects quieter voices.

Example: 12-person mixed group—three scientists, four undergrads, three fishers, two elders (one hard of hearing): each finds accessible entry via shared screen showing live hydrophone spectrogram (if available) while facilitators use hand signals and tactile drums.

Audio references for examples: to the author's knowledge, no soundweaving recordings currently exist, as the practice requires live co–creation with ocean soundscapes; this paper offers the first documented and theorized account of that process. However, the following are a few representative precursor practices that approximate elements of soundweaving:

- Paul Winter Consort. (2007). Crestone [Album]. Living Music. Soprano sax weaves with live coyotes, birds, cranes in mountain acoustics. https://paulwinter.com/crestone/
- Paul Winter Consort. (1994). Prayer for the wild things [Album]. Living Music. Whale songs interlock with sax (“Icarus”) – demonstrates marine reciprocity. Major streaming platforms.
- UC Berkeley Javanese Gamelan. (2023, April 20). Ladrang Wilujeng [Video]. YouTube. Classic pinjalan interlocking patterns—illustrates group synchrony. https://www.youtube.com/watch?v=5Tjw9qGM5RI

Recording and reflection practices: sessions are not routinely recorded to prioritize live presence and ocean agency over documentation (consistent with deep listening ethos). Post-session debriefing occurs via 10-min circle sharing. Journal prompts and group discussion translate sonic experience into articulated insights on relational ocean citizenship. Reflection outcomes align with triad progression: soundwalking builds awareness, soundsitting deepens attunement, soundweaving cultivates ethical reciprocity—scaffolding ongoing multispecies care.

### 6.3 Relational outcomes

Soundweaving advances art-science sonic work (Whittaker et al., 2024; Whittaker, 2026; Coutu et al., 2024) by framing co-creation explicitly as a pedagogy for relational ocean literacy and stewardship. Heightened vulnerability—negotiating silence amid ocean agency—co-produces trust, humility, and mutual obligation within more-than-human communities. This aligns with Levi and Peters (2024) on affective human-ocean relations beyond technocratic governance. As the triad's culmination, soundweaving rehearses geocentric ethics through embodied sonic citizenship, transforming abstract ocean literacy into lived relational practice.

## 7 Conclusion

Soundwalking, Soundwalking, soundsitting, and soundweaving form a conceptual model for relational ocean pedagogy, progressing from observational listening (soundwalking), to extended situated receptivity (soundsitting), to co-creative sonic kinship (soundweaving). Deployed sequentially or in combination, the triad offers scalable pathways grounded in deep listening and sonic relationality, transforming ecological concepts into lived aural encounters. By centering the ocean's vibrant acoustic agency, it challenges anthropocentric paradigms, positions sound as a portal for collective care, and cultivates relational ocean literacy, geocentric ethics, and ocean citizenship (Feld, 2012; Gould et al., 2021; Kimmerer, 2013; Lipari, 2014; Oliveros, 2005).
